# Longitudinal high-frequency ethnographic interviewing to simulate and prepare for intensive smartphone data collection among veterans with homeless experience

**DOI:** 10.3389/fdgth.2022.897288

**Published:** 2022-08-12

**Authors:** D. Keith McInnes, Shawn Dunlap, Gemmae M. Fix, Marva V. Foster, Jennifer Conti, Jill S. Roncarati, Justeen K. Hyde

**Affiliations:** ^1^Center for Healthcare Organization and Implementation Research, VA Bedford Healthcare System, Bedford, MA, United States; ^2^Department of Health Law, Policy and Management, Boston University School of Public Health, Boston, MA, United States; ^3^Boston University School of Medicine, Section of General Internal Medicine, Boston, MA, United States; ^4^Center for Healthcare Organization and Implementation Research (CHOIR), VA Boston Healthcare System, Boston, MA, United States; ^5^Department of Quality Management, VA Boston Healthcare System, Boston, MA, United States; ^6^Department of Health Policy and Management, Harvard T.H. Chan School of Public Health, Boston, MA, United States; ^7^Boston Health Care for the Homeless Program, Boston, MA, United States

**Keywords:** digital health tools, homelessness, veterans, user-centered design, qualitative research, smartphone, apps

## Abstract

**Objective:**

While Veteran homelessness has steadily declined over the last decade, those who continue to be unhoused have complex health and social concerns. Housing instability interferes with access to healthcare, social services, and treatment adherence. Preventing unwanted housing transitions is a public health priority. This study is the first phase of a larger research agenda that aims to test the acceptability and feasibility of smartphone-enabled data collection with veterans experiencing homelessness. In preparation for the development of the smartphone data collection application, we utilized ethnographic methods guided by user-centered design principles to inform survey content, approach to recruitment and enrollment, and design decisions.

**Methods:**

We used a case study design, selecting a small sample (*n* = 10) of veterans representing a range of homelessness experiences based on risk and length of time. Participants were interviewed up to 14 times over a 4-week period, using a combination of qualitative methods. Additionally, 2 focus group discussions were conducted. Interviews were audio-recorded and transcribed. Data were synthesized and triangulated through use of rapid analysis techniques.

**Results:**

All participants had experience using smartphones and all but one owned one at the time of enrollment. Participants described their smartphones as “lifelines” to social network members, healthcare, and social service providers. Social relationships, physical and mental health, substance use, income, and housing environment were identified as being directly and indirectly related to transitions in housing. Over the course of ~30 days of engagement with participants, the research team observed dynamic fluctuations in emotional states, relationships, and utilization of services. These fluctuations could set off a chain of events that were observed to both help participants transition into more stable housing or lead to setbacks and further increase vulnerability and instability. In addition to informing the content of survey questions that will be programmed into the smartphone app, participants also provided a broad range of recommendations for how to approach recruitment and enrollment in the future study and design features that are important to consider for veterans with a range of physical abilities, concerns with trust and privacy, and vulnerability to loss or damage of smartphones.

**Conclusion:**

The ethnographic approach guided by a user-centered design framework provided valuable data to inform our future smartphone data collection effort. Data were critical to understanding aspects of day-to-day life that important to content development, app design, and approach to data collection.

## Introduction

U.S. military veterans are at high risk of homelessness because of a variety of social, structural, and health-related factors. Through efforts of the U.S. Department of Veterans Affairs (VA) and other public and private entities the number of veterans who are homeless decreased from 74,000 to 40,000 between 2010 and 2016 (1). While the substantial reduction is praiseworthy there are persistent trends in homelessness between veterans and non-veterans and among subpopulations of veterans. Veterans make up only 6% of the U.S. population, but 11% of adults experiencing homelessness (2). Research conducted over the last three decades has also called attention to disparities within the population of veterans experiencing homelessness (VEH). For example, African American or Black veterans of all genders are overrepresented among VEH (3, 4), and are more likely to experience prolonged or chronic homelessness once an initial episode occurs (5). Other subpopulations within the veteran population that are at increased risk for homelessness include transgender veterans, who are nearly three times more likely to report housing instability than cisgender (i.e., non-transgender) veterans (6). Veterans with cognitive and behavioral health issues, such as substance use disorders or suicide attempts are 4–5 times more likely to be homeless than veterans without these conditions.

Among VEH, there is also considerable variation in the pathways leading to and out of periods of residential instability. Homelessness is often episodic, occurring once or twice for some, many times and for long duration for others. There are also many more Veterans who are at risk of homelessness because of previous life circumstances. In response to the both the prevalence of and risks related to homelessness, the VA implemented a clinical screener for providers to assess patients' housing status. This screener indicates about 300,000 Veterans served by VA are at risk of losing their housing (7). Homelessness and housing instability are characterized by frequent residential transitions, such as from transitional housing to shelter, or from doubled up with family/friend to living out of a car (8). Disruptions caused by such transitions likely contribute to this population's poor health by interfering with access to care and treatment adherence (9–13). Understanding how different experiences with homelessness intersect with disparities among veterans who are homeless or at risk of homelessness is critical if we want to provide meaningful assistance to meet their housing goals (14).

### Methodological limitations of research on homelessness

Prior research has drawn attention to a range of factors underlying homelessness among veterans (15–18). These studies have examined more distal determinants such as adverse childhood events, mental health, social support, unemployment, and housing costs (16–20). However, there is a gap in understanding of the experiences and life circumstances leading up to, during, and immediately after transitions among this diverse, veteran population. This includes in-the-moment emotions (21), behaviors (22), geographic movements (23), and changes in social support (24) which have been linked with housing stability. For example, substance use in residential programs is often treated as a rule violation that can lead to being discharged from the program and thus a transition in housing. While substance use is often attributed to the cause of transitions like this, there are likely other factors that precede the decision to use drugs and alcohol, such as feelings of loneliness or lack of social support, frustration with bureaucratic processes, discrimination, and/or a perceived lack of control over life circumstances.

Prior work on residential instability and homelessness over time has been limited by the methodological difficulty of retrospectively gathering data about distinct points of time in the past surrounding a housing transition (25–27). In part, many studies on homelessness are limited by cross-sectional research designs. These studies may be most useful for understanding major events that participants are able to recall about prior transitions in housing. However, retrospective studies are less useful for generating a nuanced understanding of the chain of events, emotions, or experiences that may lead up to a transition in housing.

Longitudinal studies with repeated data collected over time offer greater insights into patterns of residential instability and factors contributing to these patterns. There have been a few high-quality longitudinal studies conducted with individuals experiencing homelessness. For example, the 2011–2014 Australian Journeys Home is a large-scale longitudinal cohort study of persons who are homeless or at high risk of homelessness (28). The HOPE HOME study based in Oakland, California, US is a longitudinal study of older persons who are homeless (29). There are also several longitudinal studies focusing on specific conditions or risks, such as Roy et al.'s study of HIV risk among a cohort of youth experiencing homelessness in Montreal, Canada (30, 31). Many of these studies have complicated findings from cross-sectional studies that identify risk factors such as substance use, unemployment, and mental illness as predictors of homelessness (32). Nevertheless, even when people experiencing homelessness are surveyed every 6 months, the ability to retrospectively recall and understand what may have led to an event 4 months ago, for example, is still limited (33). Gathering in-the-moment/real time data about emotions, social interactions, experiences with bureaucratic systems and processes, changes in health and mental health, and other experiences that come into play in the hours and days before a transition in housing occurs may provide insights that can be used to inform the development or tailoring of homeless prevention and intervention resources (34).

### Mobile technologies as a research tool

Mobile technologies, such as smartphones, have the potential to aid in the collection of real- or near real-time information about the sequence of events leading up to and immediately after housing-related transitions. These increasingly ubiquitous technologies may help identify “early warning” signals based on self-reports of mood, activities, social support and activity spaces (constructed from passively collected GPS data) that may presage increasing housing instability, a homelessness episode, or a major health event. However, technology enabled studies conducted with individuals experiencing homelessness are few, in part because of concerns about their feasibility. Recent research has shown that mobile phones are commonly used by people experiencing homelessness (35, 36) and that these devices are used for a broad range of purposes (35, 37–41). This has led to increasing interest in studies that explore how to deliver services *via* smartphones and meet the differing needs of this population (18).

Fewer studies have made extensive use of smartphones to facilitate the collection of real time data from people experiencing homelessness, such as brief surveys that are transmitted *via* email, text, or application (“app”) (42–44). Other tools available on smartphones such as global positioning system (GPS) also have had limited use in research (36–38). For example, GPS-enabled smartphones may be able to provide information about the mobility patterns, suggesting linkages between spatial context, day-to-day experiences and emotions, and pathways into and out of homelessness (45, 46). Researchers increasingly recognize the potential of smartphones for real time data collection in populations at risk of or experiencing homelessness (45, 46). However, the utility of studies collecting data *via* smartphone, depend on generating interest in participating and being able to ask the right questions at the right time.

### User centered design

Principles of User Centered Design offer guidance throughout the phases of designing, developing, evaluating, and refining a product, such as a data collection app for research purposes. At the heart of the process is a deep understanding of the users, the contexts or conditions of use, and factors that might influence tasks associated with product use (47, 48). Applied to a research study, cultivating an understanding of potential users or participants requires learning what is relevant and important to ask in relation to the central research question(s). Learning about the contexts of users' lives can provide valuable information for design and use features. Eliciting feedback on user preferences and potential challenges or limitations associated design features can improve the completion of tasks and activities associated with the product. Investing time early in the research process, to learn about the study population in relation to the data collection, product or tool is critical to the conduct of meaningful and useful research.

This paper draws on data collected during the formative phases of a larger research initiative to engage VEH in longitudinal research studies using smartphone applications or “apps” to facilitate real-or near-real time data collection. The formative phase used an ethnographic case study approach, guided by user-centered design principles, to gain a nuanced understanding of the daily lives of VEH in one urban geographic area of the United States. Specifically, we sought to learn about factors influencing transitions in housing that might be amenable to capturing through a smartphone app. We sampled veterans to include variation in types of homelessness (chronic, recent, at risk) to gain insights into how these different circumstances might influence use of smartphones and other related technologies (e.g., computers, tablets). Finally, we explored factors that could influence tasks associated with smartphone-aided data collection, ranging from concerns with privacy and confidentiality to design considerations (e.g., reminders, font size). Our formative ethnographic work is being used to guide the content, design, and approach to data collection *via* a smartphone app in the next phase of our research.

## Materials and methods

### Overview

**Figure 1:**
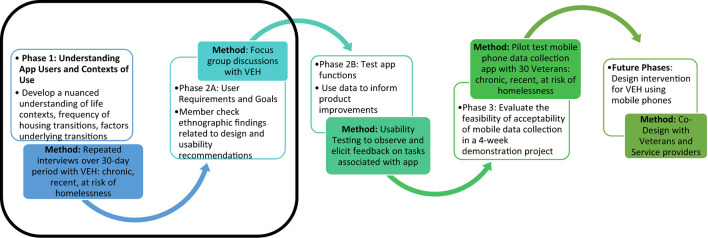
Ethnographic-informed user-centered design data collection app. *Black box represents phase of the study reported on in this manuscript.

The formative phase of this larger study entailed the use of an ethnographic case study methodology focusing on the experiences of a diverse sample of VEH, including those who were at risk of homelessness, newly homeless, and experiencing chronic homelessness. A case study is a detailed examination of a single or small number of individuals, sites, or event aimed at generating context-dependent knowledge that is useful for developing theories about social phenomena of interest (49, 50). In this study we sought to learn about the relationship between events, activities, and emotional states related to transitions in housing and health, and the day-to-day use of smartphones and technology. A mix of long and brief qualitative interviews and observations conducted over multiple weeks allowed for exploration of historical and current factors that influence transitions in housing (Phase 1). Phase 1 interviews and Phase 2 focus groups also collected information on design and methodological considerations. User Centered Design principles (47) and the Uniform Theory of Adoption and Use of Technology (UTAUT) (51) guided inquiry into factors that may influence willingness to engage in and participate fully in a smartphone-enabled data collection study. Figure 1 provides an overview of the study phases and goals.

### Setting

The U.S. Department of Veteran Affairs (VA) provides healthcare and aligned social services for over 9 million military veterans annually. It is the largest integrated healthcare system in the U.S., and one of the largest providers of services to persons experiencing homelessness. This study was conducted in two locations in the northeast region of the U.S. Site 1 is one of the VA Healthcare System's 141 medical facilities, located about 15 miles north of a major city. It provides a range of health and social services to veterans living in suburban and rural areas. The second site was located in the center of a dense, urban area. Here we recruited veterans from a multi-service residential program serving veterans who are experiencing homelessness and at risk for homelessness. The [City] Healthcare for the Homeless Program, which offers low barrier, low threshold services in the metropolitan area, also referred veterans in their care to the study.

### Participant sampling and recruitment

A convenience sampling strategy was used to recruit ten participants with a range of experiences with homelessness, including chronic homelessness, a recent onset of homelessness, or at-risk for homelessness. The study team purposely recruited at least three veterans from each category to learn from people with diverse experiences with housing instability. For the purposes of this study, we defined these three groups as follows: *Chronically homeless* means having a pattern of multiple episodes (or a single long episode) of homelessness over an extended period of time (i.e., a year or more). This could be, for example, a person who lives primarily on the street and/or in a short-term shelter for long periods of time. *Recent onset* means newly homeless, with the period of homelessness having begun in the last 6 months, but not having a history of being chronically homeless. *At risk* refers to being at imminent risk of losing one's housing. At risk was determined in one of three ways: (a) a case manager knows that an individual is about to lose housing or is likely to lose housing; (b) a Veteran seeking Supportive Services for Veteran Families (SSVF) prevention services from a community based organization; or (c) a veteran was identified as at risk in response to a health provider's use of the VA Homelessness Screening Clinical Reminder which asks if a Veteran is concerned about not having housing in the next 60 days. These categories and definitions were adapted from the U.S. Housing and Urban Development's annual Point-In-Time count and report to the US Congress (52).

A multi-pronged effort was used to recruit participants. For the Phase 1 longitudinal case studies, the study team hosted informational sessions and posted flyers at residential programs in Site 1 and 2. Those that expressed interest were approached by study team members during informational sessions or later contacted by telephone. They were then screened for their current homeless experience (chronic, recent-onset, and at-risk) to confirm that they met eligibility criteria. Participants who met criteria provided written informed consent and assessed for cognitive functioning using the Montreal Cognitive Assessment (53). The results of the assessment were not used as part of the study criteria, but to identify the potential need to spend more time reviewing informed consent procedures or potentially tailor the approach to collecting interview data.

Phase 2 focus group participants were recruited from Site 2. Focus groups took place at a nearby VA hospital and on-site at the residential program. Only veterans with recent onset and chronic homelessness were included in these group discussions. In part, this was due to challenges identifying and recruiting veterans who were at-risk for homelessness in Phase 1. Through rapid qualitative assessment of Phase 1 data we found few differences in the themes emerging between those at risk and those with recent onset of homelessness. We therefore used the focus groups to collect feedback on topics important to the app design and research approach.

### Methods: Ethnographic case study

Phase 1 included repeat interviews over a 4–6 week period. This study period is consistent with the planned timeframe for the Phase 3 pilot study of smartphone-enabled data collection and offered a proof of concept for engagement in this later work. Four different types of interviews were conducted during this period (see Table 1): (1) a baseline, historical interview that lasted between 60 and 90 min; (2) Rapid Qualitative Interviews focused on a “special topic” of interest to the study that lasted ~60 min (RQI-L); (3) a general Rapid Qualitative Interview, twice a week, that lasted ~15 min (RQI-S); (4) a final interview to obtain feedback on participation in a multi-week study. The baseline interview was designed to gain a deeper understanding of participants' housing history, using a modified Residential Time-Line Follow Back Inventory (54, 55). Key social relationships, military experiences, and employment opportunities that may have influenced each individual's life course were explored. Demographic information was also collected and recorded on a short survey form during this initial interview. Longer “special topic” interviews (RQI-L) provided an opportunity to gain insights into three different topics over the course of participation: (1) physical and mental health (e.g., perception of overall health, experience with physical and mental health conditions and the extent to which they impact everyday life, perceptions of the impact of health conditions on housing stability); (2) Access to and use of social services (e.g., places participants go for financial, logistical, and/or social support to meet needs, facilitators and barriers to accessing different types of support, and perceptions of how support influences housing stability; and (3) Use of technology (e.g., type and uses of phone, including common apps and phone features). In between the longer special topic interviews, participants were called ~2–3 times each week for short interviews (RQI-S) to explore (a) how were feeling, (b) where they slept the night before, (c) any changes in their lives since the last conversation. These were meant to be brief conversations (~15 min) to identify and document any abrupt changes in participants' lives. At the end of the study period, a final 60-min interview was conducted to obtain feedback from participants on the experience of engaging with a research team over a prolonged period of time. These interviews also provided an opportunity to ask their opinions about the most important things to ask on a regular basis that might influence stability in housing, how to ask about sensitive topics like substance use, and about potential concerns with answering questions through a smartphone app. Experienced qualitative researchers conducted all interviews, with one interviewer assigned to follow each participant, to build rapport and knowledge. The interviews were conducted in a location of the participants' choosing, including a private room in Site 1 or Site 2, outdoors, or *via* phone.

**Table 1:** Schedule of phase 1 ethnographic data collection.

**Week 1**				**Week 2**			**Week 3**			**Week 4**		
**Baseline**	**RQI-S**	**RQI-S**	**RQI-L**	**RQI-S**	**RQI-S**	**RQI-L**	**RQI-S**	**RQI-S**	**RQI-L**	**RQI-S**	**RQI-S**	**Final**
Interview 1	Interview 2	Interview 3	Interview 4	Interview 5	Interview 6	Interview 7	Interview 8	Interview 9	Interview 10	Interview 11	Interview 12	Interview 13

Phase 2 entailed two focus groups with veterans who met Phase 1 inclusion criteria. The first focus group had two participants and the second had seven participants. These discussions provided an opportunity to build on the Phase 1 findings and further explore how to recruit, enroll, and retain VEH in a future study such as ours. The focus group participants were asked for their general perceptions of taking part in a research study that uses a smartphone app for data collection and recommendations regarding how to: (1) introduce and explain a study that uses the collection of data through a smartphone app, (2) explain the ways in which privacy and confidentiality will be maintained, (3) ask about potentially sensitive topics, such as substance use and relationship conflict, and 4) assess capacity to participate using a smartphone app. Participants were also provided a brief questionnaire to obtain specific information about their access to and use of technology generally, and specifically smartphones. Focus group discussions lasted ~60 min and were facilitated by one of the anthropologists on the study team.

### Ethical considerations

Study procedures were approved by the VA Bedford Institutional Review Board. Participants provided written informed consent and received up to $185 USD reimbursement over the course of the study ($25 for the baseline, follow-up, and long RQI interviews, and $10 for each brief RQI interview) for Phase 1. Participants in Phase 2 received $25. Randomly generated ID numbers were assigned to participants to ensure confidentiality. No personal identifying information were used in the audio-recordings.

### Analysis

#### Quantitative data analyses

Demographic data were entered into a Microsoft excel spreadsheet and analyzed to generate descriptive information. The participants' ages were described using median and interquartile ranges and the descriptive data was summarized using counts, frequencies, means and percentages.

#### Qualitative data analyses

All interviews (one-on-one and focus groups) were professionally transcribed verbatim. Data were analyzed using a Rapid Assessment, Response, and Evaluation (RARE) approach (56), which uses a multi-disciplinary team to collect different types of data that can be synthesized and analyzed iteratively and efficiently to generate an understanding of critical health and public health issues. Each participant in Phase 1 comprised a “case” and had a portfolio of data from their multi-week study period. Analysis entailed attention to both the cumulative story and key changes that might impact housing transitions or health. Analysis of qualitative data began with interviewers preparing an analytic memo summarizing key impressions, immediately following the baseline interview to summarize information from the qualitative and quantitative questions (e.g., residential history, important relationships, and circumstances currently affecting housing stability, etc.). This memoing processes was then built on over the course of data collection, adding analytic memos to the baseline document, capturing key learnings about factors influencing physical and mental health, social relationships, use of social services, and use of technology. Adjacent to these open-ended memos, which capture information from participants and analytic insights, each interviewer identified categories of information (e.g., events, relationships, and perceptions) that were salient in participant's lives.

Once the transcripts of each interview became available, the interviewer reviewed the transcript and added detail to the analytic memos, including illustrative quotes. A standardized template was created to systematically summarize data related to transitions in housing and health for each participant. The summarized information was examined to identify patterns, common concepts, and emerging ideas about current events and experiences that influence transitions in housing and health. The lead interviewer and one other team member paired up to review the analytic memos and the resultant summaries for each template to assure consistency. These were then discussed by the full team. Phase 2 focus group data was analyzed using a similar process. Templates for these interviews focused in specifically on recommendations for improving the introduction of the study and approach to data collection *via* smartphone app. We also captured information related to recommendations for asking about sensitive topics *via* an app-based survey, such as substance use or loss of housing.

Data summaries were reviewed to both identify factors related to historical and current housing transitions, focusing specifically on understanding the chain of events that lead to transitions. Similarly, we explored the contexts or environments of participants' lives, which influence the ability to use and maintain smartphones and other technologies. Building off Phase 1 findings about how technology is used to navigate and access resources, social support and connection, and other uses, Phase 2 focus group transcripts were reviewed to dive deeper into specific topics relevant to smartphone use and issues related to our future data collection effort *via* an app that are important to build into the design and content of questions.

## Results

We collected ethnographic data from 10 veterans who met inclusion criteria (chronic, recent, at risk for homelessness). In addition, 9 veterans actively experiencing homelessness (chronic or recent) took part in focus group discussions. Across 10 veterans who participated in Phase 1 ethnography, we had 45 transcribed interviews and dozens of brief notes from RQI-S interviews. The majority of participants participated in all data collection activities; 2 did not complete the final debrief interview and 1 was lost to follow up after completing 2 of the longer interviews. The participant lost to follow up was unsheltered and did not have a mobile phone at the time of enrollment. We provided him with two phones during the short time he was engaged in the study, both of which he reportedly damaged.

Below we provide an overview of key insights from our ethnographic research and the influence of these insights on the development of our Phase 3 pilot study. Following a description of the sample, we present findings related to the ubiquity of smartphones and uses among the sample. We then describe common circumstances and events observed or reported on during the study period and how they may influence transitions in housing. Finally, we highlight factors that may influence smartphone-enabled data collection with a population of veterans who are experiencing or at risk of homelessness. After each section, we provide an overview of how these key findings are influencing decisions about the content, approach, and design of the smartphone data collection app we are developing.

### Sample characteristics

Table 2 provides an overview of key demographic characteristics of our Phase 1 and 2 samples. Although small, the sample mirrors the demographic characteristics of VEH in the region where the study was conducted. The sample included nine men and one woman. They ranged in ages from their 30–70s with most being between the ages of 40 and 60 years. Four were African American and six were white. The majority (*n* = 6) were staying at Site 2, a housing program and multi service non-profit for veterans experiencing homelessness. Several (*n* = 3) were in a VA domiciliary program (Site 1); one was unsheltered, living on the street. Half (*n* = 5) reported experiences of homelessness or unstable housing as a child. Less demographic information was obtained from focus group participants. All 9 focus group participants were categorized as being either more recently homeless or chronically homeless; no veterans who were at risk for homelessness were able to be recruited for these discussions. Among the nine participants, all (9 of 9) were male. The majority (6 of 9) were white and 3 were black or African American.

**Table 2:** Demographics of Phase 1 and 2 participants.

	**Phase 1 interviews (*n =* 10)**	**Phase 2** **focus groups (*n =* 9)**
**Gender**, ***n (%)***		
Males	9 (90%)	9 (100%)
Females	1 (10%)	
**Age range, years**, ***n (%)***		
30–40	1 (10%)	
40–50	3 (30%)	
50–60	4 (40%)	
60–70	2 (20%)	
**Race/ethnicity**, ***n (%)***		
White	6 (60%)	6 (67%)
Black / AA	4 (40%)	3 (22%)
**Type of homelessness experience**		
Chronic	5 (50%)	
At-Risk	3 (30%)	
Street	1 (10%)	
**Current housing type**, ***n (%)***		
Shelter (Veterans only)	6 (60%)	9 (100%)
VA Substance Use Treatment Facility (Domiciliary)	3 (30%)	
Other	1 (10%)	
**Number with childhood housing instability**, ***n (%)***		
Self-reported	5 (50%)	
**Number with Military-Related Trauma**, ***n (%)***		
Self-reported	3 (30%)	

### Frequency of residential transitions during study period

We captured variation and changes in housing status across the 10 participants over the 30-day study period. The data collection period for Phase 1 of the study was designed to last ~30 days, which is the length of time proposed for the Phase 3 pilot study that will entail smartphone-enabled data collection. During this formative phase, half (5 of the 10) of the study participants had transitions in housing. Three of these participants moved from transitional housing or shelter into permanent housing. One of these participants reported not wanting to work with case managers at the transitional program he was in because there were too many rules and requirements to follow. He preferred and successfully did find housing on his own. The other two moved into a Single Room Occupancy unit that was managed by the transitional housing program they were living in when first enrolled in the study. Two participants had to leave their residential treatment and shelter programs due to rule violations. One was found with an over-the-counter medication in his room that was not reported upon entry into the program. During the study period, he moved to one short term transitional housing program before finding another treatment program. He was able to maintain a full-time job throughout these transitions. The other was asked to leave after getting into a fight with other residents. This was at the end of our study period and we do not know where he transitioned. Two other participants were very close to moving out of a transitional housing program and into their own subsidized apartment, but this did not occur until after the study period ended. Three of the remaining participants were interested in finding their own housing at some point in the future, but for a variety of reasons (e.g., needed to feel more stable in recovery) this was not a priority at the moment. Only one participant indicated that he was not looking for more permanent housing. This individual was unsheltered and lost to follow up during the study period.

Besides these major transitions, such as moving out of a transitional or shelter program and into more permanent housing, the majority of participants who were homeless at enrollment stayed in the same residential program each night. A few participants noted that they would occasionally “take a break” from their temporary housing program to sleep in a hotel for a night or two. This allowed them some privacy, a greater sense of safety, and a better night's sleep. These “respites” typically happened around the beginning of the month, when VEHs receive their disability or other benefit payments. There was also a seasonal component to the “first of the month” phenomenon, which one participant referred to as the “curse of the first.” During the Spring and Summer months in New England, the consequences of losing one's housing were perceived to be less concerning because it is warm enough to sleep outside. A few participants noted they were more likely to take risks with their shelter or transitional housing bed (e.g., stay out past curfew) during these warmer months. However, regardless of season, most participants surviving on a fixed income (i.e., benefits) reported that they have dwindling income to live on over the course of a month. As noted below, this often coincided with fluctuations in levels of stress, frustration, and depression.

### Implications for phase 3 pilot

#### Content

A 30-day data collection period may be long enough to capture real time transitions in housing for at least a subset of participants. Although we planned on frequently asking information about where a person slept the night before, we may consider adding a question to explore whether changes were temporary/short-term or permanent/long-term. Additionally, the events surrounding a transition were relatively stressful and time-consuming for participants. Capturing fluctuations in moods and behaviors through ecological momentary assessments (EMA) will be critical to learning how participants perceived and manage these changes.

#### Approach

There was some temporary loss of responsiveness to interviewers during the week leading up to and following a transition. Although Phase 1 data collection was more time intensive for participants (i.e., repeated interviews, lasting 15–60 min), we may anticipate a drop in participation in daily data collection surveys during a housing transition. This has led to thinking about ways to accommodate a busy period by either shortening surveys or allowing participants to pause participation or extend participation to allow for 4 full weeks of data collection.

#### Design

Interviews indicate that there may be fluctuations in activity spaces over the course of a month and by season (at least in places with highly seasonal weather patterns, like New England). Those who mentioned taking a break from shelters by staying in a hotel indicated that this entailed taking public transportation to an outlying suburb of the city where hotels are less expensive. Similarly, having more money at the beginning of the month may mean they are able to get to and purchase goods in locations further away from their current housing. GPS features on smartphones will be useful for exploring fluctuations over the course of a month. We will also want to ensure that recruitment of participants happens across seasons to explore climate-related variation in activity spaces.

### Factors underlying fluctuations in housing

Most participants described a long history of residential instability that began in childhood. These experiences continue to influence their lives, particularly their physical, mental, and behavioral health, social relationships, and income. Below we highlight how historical experiences continue to influence participants' everyday lives and housing stability.

#### Trauma

In baseline interviews, the majority of participants described their housing instability as starting in childhood. Abuse and neglect, familial substance abuse, and frequent moves due to divorce and/or poverty were common. Joining the military at age 18 was perceived to be one of the few ways for participants to change their circumstances. However, entering military service with fairly significant and unaddressed trauma histories often posed more challenges for participants. Many were discharged before completing their service and returned to similar circumstances, routines, and habits. Residential instability shortly after separation from military service was commonly reported.

A few also identified traumatic incidents that occurred during their military service, such as military sexual trauma, which continues to affect their mental and physical health into the present day. As one black male participant noted, the sexual trauma he experienced while in the service filled him with shame and guilt. There was little recognition of military sexual trauma in the 1980s when he separated from service. Similar to others with histories of trauma, he spent nearly two decades coping with drugs and alcohol, which affected his personal relationships, employment, health, and housing.

Participants' prior trauma experiences are important to understand as they impact their housing transitions and residential instability in direct and indirect ways. In the Baseline and RQI interview focused on health, participants described how symptoms associated with trauma, such as chronic nightmares, hyper vigilance, depression, and anxiety, interfere with one's ability to engage in everyday activities. For example, one participant who was unsheltered and living on the streets described in his first interview a broad range of social support services he avoids because they are full of “skinners and rippers” (people who molest children and rape people). He was molested as a child and continues to feel the reverberating effects of these experiences more than 50 years later. For him, isolation and detachment have become strategies for self-protection. His avoidance of social and health services was on the extreme end of the spectrum. Most were accessing some support services, mainly housing support. A few participants were engaged in individual and/or group therapy for trauma and substance use. These participants generally felt that these supports provided a buffer against recurring trauma symptoms, while also offering them tools to manage symptoms when triggered.

In their everyday lives, participants also faced a broad range of challenging and often traumatic events. Being mugged and having belongings stolen was common across participants who were chronic and recently homeless. Many described their living situations as constantly chaotic and stressful, requiring hypervigilance.

*It's not really tents. I just pull a tarp over me. I am a Marine, we just lay on the ground and pull a tarp over…It's camping yeah but it's incognito. That way you don't get knocked in the head. I got knocked in the head many times. And they steal your stuff. You got to sleep with one eye open. It's kind of like combat. 24/7. (V-104, chronic)*

Violence on the street and seasonal cold weather led many to seek out shelters and transitional housing programs. While these locations were relatively safer, violence, verbal harassment, and theft within group living environments was commonly reported. For many, the unpredictability of these environments and hypervigilance was exhausting.

### Implications for phase 3 pilot

#### Content

The intensive longitudinal approach to data collection shed light on the potential relationships between prior experiences of trauma and current transitions that impact housing stability. Among participants with these experiences, fluctuations in stress, anxiety, frustration, and depression emerged as critical to capture on a daily basis. Negative changes in these emotions have a strong ripple effect for those with histories of trauma as they can be triggers for coping mechanisms such as drug and alcohol use and violence (verbal and physical). We will also have an opportunity to learn if engagement in professional support or treatment serves as a buffer to these stressors, including activities or strategies that are used to manage challenging situations.

#### Approach

A second implication of understanding participants' prior histories of trauma and military experience is related to trust. Participants with prior histories of trauma were more likely to note throughout our engagement that they do not trust many people, if anyone at all. In our final Phase 1 RQI-Long interview and focus group discussions, participants noted the importance of taking time up front in the Phase 3 study to build a relationship with participants. They specifically recommended having the study be introduced in person, with a clear explanation of study purpose and how the data would be used. They also recommended having an initial long interview at baseline so that participants get to know more about the kinds of questions we have and who (study team) will be looking at their data.

#### Design

With high levels of trauma anticipated among the study sample, we are also looking into principles of trauma-informed app design (57, 58). Although a new concept for User Centered Design practitioners, there are key principles that are aligned with feedback from Phase 2 focus group participants.

*You gotta put all these questions into short-form. Make it quick, to the point. If you drag it out, you'll lose the concentration of the servicemen. With post-traumatic stress, you're looking around all the time, you get that in the streets too cuz you're worried about that guy stabbing you, stealing your wallet… None of these long-drawn-out things that we need to read forever. Get it to the point and then check it off. I recommend that for all men and women who have been to war and have trauma in their life. (Focus group 1 participant)*

Key principles of trauma-informed design include: (1) take time to build trust with participants, (2) be clear about the purpose of the study and how information will be used, (3) have a recognizable logo for the app so that when push notifications appear people know they can be trusted, (4) be mindful of the cognitive burden associated with each question and minimize complexity and volume of response options, and (5) offer choice to participants to not answer questions they are uncomfortable with and to provide open text fields if more information wants to be shared.

### Physical, mental and behavioral health

Nearly all participants reported managing a range of physical, mental, and behavioral health issues. Chronic physical conditions included hypertension, diabetes, chronic obstructive pulmonary disorder, cancer, and HIV/AIDS. Mental health conditions commonly reported included post-traumatic stress disorder, anxiety, depression, and bipolar disorder. Behavioral health concerns were largely linked to alcohol and drug use.

Participants who experienced chronic homelessness had the greatest number of health concerns and struggled the most to manage them. These individuals were more likely to report a lack of trust in healthcare providers and poor prior experiences trying to access care. At the other end of the spectrum, those who were at risk of homelessness tended to be in treatment programs and receiving the healthcare they wanted. This group and the recent onset of homelessness group also tended to be younger and had fewer reported physical health problems.

The longitudinal nature of our study allowed us to capture a number of instances when physical, mental, and behavioral health issues interfered with housing or other goals that participants had on a day-to-day basis. One participant's experiences demonstrate the intertwined nature of housing, health and poor adherence. This participant had several complex chronic conditions, including diabetes, high blood pressure, asthma, neuropathy, and an alcohol use disorder, found it difficult to leave her room at the shelter on many of the days we spoke with her. On some days she missed appointments because she was not feeling well or she was unable to figure out how to get to her medical appointment on foot. Missed appointments meant that she was not able to communicate with her care team as her health was deteriorating. It also meant that she missed her substance use treatment appointments, resulting in a lapse in medication she took to deter her alcohol use. In addition, her fiancé who was staying in a different shelter became increasingly agitated and verbally violent over the phone on some weeks when she was not able to meet him and provide him with money. With others in the shelter drinking, she found herself joining them on some days to cope with the stress. Under these circumstances, it was difficult to do things she needed to do to complete her application for subsidized housing, such as get her state identification card and complete paperwork.

Unaddressed mental and behavioral health issues were associated with a bundle of problems that influenced housing stability, including job loss, incarceration, and strain in personal relationships. Specific substances of concern included alcohol, heroin, and cocaine. Marijuana use was frequently mentioned, but not generally perceived to be problematic. Several talked about their substance use as a way of managing their mental health needs. Veterans with more extensive histories of homelessness also talked about using drugs and alcohol to cope with symptoms of post-traumatic stress, which was frequently triggered as their current circumstances required hyper-vigilance (“you can never let your guard down”), an ability to handle unpredictability (“it's total madness here”), and a tolerance for challenging social dynamics. A few were new to mental health treatment and just beginning to reflect on their personal pattern of drug and alcohol use as a way to self-medicate and manage symptoms.

*I smoked pot. That's what I was using to self-medicate my bipolar before I was on medication but, and then alcohol, I drank alcohol, but I wouldn't say it's like a substance abuse problem. You know, it's more me trying to manage my bipolar and the times I've had too much to drink, it's usually I'm in a manic state and trying to suppress it, and it just like, it comes out, you know, and I think a lot of, you know, the problems I've had with alcohol stem from me being bipolar*.

*(Vet-315, at risk)*

Vet-315 had recently been released from jail and was in a treatment program where he was getting the mental health care he felt he needed. He was optimistic that the medications he was on to help manage his bipolar disorder would result in not feeling the need for other drugs. A few participants indicated their choices were to isolate themselves as much as possible to avoid others or to give in and join them. Although most wanted to isolate themselves and do what they needed to move into more permanent housing, we observed fluctuations in mental health, particularly depression, over the course of a month. Worsening physical health, repeated bureaucratic delays, and interpersonal conflicts were among the common “flashpoints” that led some participants back to drug or alcohol use.

Most participants described abstaining from drugs and/or alcohol for periods of time. In general, these were times when they had more stability in their housing and thought of themselves as doing pretty well. However, many also experienced changes in their circumstances that led them back to drug and alcohol use, which set off a chain of events that affected their housing stability. For many participants in our study, the COVID-19 pandemic was a recent example of this fluctuation. For example, one participant who was at risk for homelessness was working two jobs and living in his own apartment prior to the pandemic. His job gave him meaning and purpose in his life and kept him busy. Shortly after the pandemic caused a major shut down of many non-essential services, he was laid off one job and then his second one shortly after. He suddenly found himself isolated with a lot of time on his hands. His social network became much smaller, comprised largely of other Veterans who were living in his housing complex. They were “all drinking and doing drugs.” Without a “purpose and reason to get out of bed in the morning” he started using again to fill the void. Over the course of 6 months, his use escalated and led to several substance-related hospitalizations. He was at risk for losing his housing and entered a substance use treatment program, which is where he was when he enrolled in the study.

A third group indicated that they do not see their alcohol or drug use as a problem at all. They described using these substances to either deal with the monotony of everyday life or to manage the chaos that is around them at a shelter or transitional housing program. They push back on the notion that if you have a drink or two at night you have a problem or that this is the underlying reason for homelessness. This perspective came up most strongly in focus group discussions about how to ask questions about sensitive or stigmatized issues through an app. Participants pressed group facilitators on why questions about substance use were important and how the information would be used if participants answered them.

### Implications for phase 3 pilot

#### Content

Physical, mental, and behavioral health conditions are intricately linked to fluctuations in participants' mood, sense of hope for change, and motivation to work toward housing and other goals. In baseline interviews it will be important for the research team to gain an understanding of all the health conditions participants are living with and their perceptions of how well they are able to manage them. Throughout the pilot phase, we will include brief questions about perceived physical, mental, and behavioral health status (e.g., How is your physical health today? Did you use drugs today?) and the extent to which they believe their health impacted the ability to do the things they wanted to do. We will use Ecological Momentary Assessments to understand fluctuations in mood throughout the day. At the end of each day we will also ask about engagement in health services (e.g., services accessed, appointments missed) and the extent to which participants feel they received the professional support they needed to help manage health conditions.

The salience of drug and alcohol use and the ways in which directly and indirectly influenced participants' housing stability makes it an important topic to ask about in the Phase 3 pilot. However, participants expressed hesitation with sharing information through an app about their substance use. Among some participants, their experience is that when they mention using drugs or alcohol to a professional health or social service provider, it tends to become the lens through which all other circumstances and behaviors are understood. They are labeled as “drug addicts” or “alcoholics” and substances become the main focal point for services.

*We don't want to be stereotyped as having “that issue” [drug or alcohol problem] bringing us down to where we are, cuz I know a lot of people that don't have issues at all that are lost and homeless due to circumstances… Because it [being labeled] really affects you if you're homeless… (Focus Group 1)*

Others expressed some legal and privacy concerns with sharing detailed information about drug use in particular. This was particularly true for participants who were on probation or parole. One other potential barrier to asking about substance use was related to participants' readiness to admit it. For example, one participant noted that he would have no problem answering questions about his substance use because he was not currently using drugs or alcohol. When asked to think about a time when he was using and the extent to which he would feel comfortable answering the same set of questions, he noted “*that would be a different story*” *(V-314, At-Risk)*. For this participant, the shame of using drugs and alcohol usually leads him to feel reluctant to share this information with others until he is ready to stop again.

#### Approach

As already mentioned in the section on trauma, investing time during the initial study enrollment period in getting to know participants' life experiences, circumstances, and perspectives in relation to their health will be important. Not only will it inform our interpretation of daily survey data but will also give participants a chance to learn who we are as a research team and form an opinion about the importance of the research. If we are able to do this well, both through a thoughtful informed consent process and a meaningful baseline interview, we may improve our chances of participants responding to questions about highly stigmatized or sensitive topics, such as drug and alcohol use, trauma, and mental health challenges.

Our team discussed how to ask questions in ways that are psychologically safe, non-judgmental, and low burden. In addition to using neutral, person-centered language, we are also considering what we actually might need to know about things like drug and alcohol use. For many participants, it may be sufficient ask two questions about drug and alcohol: (1) use of drugs/alcohol today, and (2) perception of if use (or not) influenced their ability to do what they wanted to do. For each question, we will also include a “decline to answer” choice option.

Finally, findings from this formative phase have led us to consider adding questions to our enrollment screener to help identify individuals for whom participation in a smartphone-enabled study would not be appropriate for participation. Examples of individuals who may not be appropriate include those who believe they are under constant government surveillance through technologies like smartphones or who have a deep mistrust of government institutions (such as the U.S. Department of Veteran Affairs).

“*We don't like to be tracked, we don't like to be watched, basically most of us are a little paranoid. We don't want anyone asking where I'm going, what I'm doing.” (V-104, Chronic)*

The Phase 3 pilot study has a relatively small sample size (*n* = 30). Given the sensitivity of some questions we want to ask about (e.g., mental health and substance use) and data collection features we want to try out through the smartphone app (e.g., GPS location data), we want to assess our “proof of concept” with a sample of individuals who are willing to participate from the outset.

#### Design

As a research study, the research team is required to ensure that all data collected through the smartphone app is securely obtained and stored on HIPAA compliant servers, with access only granted to members of the research team. In addition, the app will use end-to-end encryption to send survey, GPS, and other data to the HIPAA compliant server. Finally, we have obtained a Certificate of Confidentiality from the National Institute of Health to comply with Section 2012 of the twenty-first Century Cures Act (42 U.S.C. 241). This adds a layer of protection to our data to ensure that identifiable data cannot be provided to legal or other non-research parties. While these precautions are relatively standard for a research study, it is important that we are able to clearly communicate these precautions and educate participants on the many ways we will protect the privacy and confidentiality of the information they provide.

### Social relationships

There were three types of social relationships that participants described as influencing their everyday lives in both positive and negative ways. The most influential were family and friends, followed by professionals, such as case workers and clinical providers. Participants were also influenced by the people that lived in residential or transitional housing programs, whom many referred to only as “acquaintances.” The majority of participants relied almost exclusively on their smartphones to maintain relationships with family members and professionals, with some (mostly older participants) preferring to talk by phone, while others preferred text or video applications. Phone theft and/or phone damage was a common occurrence and a major stressor for participants. The loss of a phone often meant the loss of important information such as contacts, upcoming appointments, and electronic documents. Participants who reported the fewest personal and professional relationships were also more likely to report the greatest frequency of phone loss over the last year.

Relationships with family members varied significantly across the sample. On one end of the spectrum, there were two male participants who reported no contact with family members, including siblings and children. These participants were chronically homeless and described extensive instability and trauma from an early age. A second group of participants (*n* = 6) described maintaining some relationship with family members, mostly their adult children and/or mothers. Some in this group reported that these relationships can be emotionally supportive, but no one reported that they offered financial or logistical support. On the other end of the spectrum, two participants reported they frequently speak with family members, who provide substantial emotional support. One female participant reported the greatest contact with family members, connecting with her mother and kids nearly every day. Participants noted that they were better able to maintain contact with family members when they were housed. These connections diminished when they begin using drugs or alcohol, experience a downturn in their physical and emotional health, and/or lose housing.

Participants in our sample frequently described connections to other individuals in their day-to-day lives that provided social support. They included other veterans, romantic partners, social service workers, and sobriety sponsors. For some, these relationships were a key source of support as they navigated homelessness. Some described receiving access to hot showers, food, laundry, and temporary sleeping accommodations, as well as physical, emotional, and spiritual support.

“*She [friend who was once homeless with participant] helped me out as she could. She let me take showers, let me spend the night over there. She fed me, hooked me up with some weed… my own family members would ride by me… But people who actually help me, that was there for me, that call me [and ask] How you doin?, How's things goin?, What do you need?, Are you all right?, There's only like four or five people that do that.” (V-102, Chronic)*

A few participants also described forming relationships with other veterans experiencing homelessness. These veterans were often living in the same transitional housing or treatment program. Similar to social service providers, these relationships provided information about potential housing opportunities, financial assistance, sobriety support, and support for trauma resulting from military service (e.g., PTSD, military sexual trauma):

“*Veterans tell me this is a place you can go to if you got an unpaid bill and you can't pay it, they might help you out, stuff like that… they might have a resource where if you owe an electric bill from an old apartment and you're trying to move to a new apartment, they might have funding…” (V-315, At-Risk)*

During the study period, we also observed frequent turmoil and disruptions in these relationships. One participant broke off his engagement with a woman who he described in the baseline interview as “my number one” (*V-101, Chronic*). Several others talked about arguments they had with romantic partners that led to them to feeling more depressed and isolated within the span of just a few days. Some personal relationships were particularly difficult to navigate. This was especially true among participants who were working on making changes so that they could obtain housing, employment and/or address health concerns. For example, one participant with a significant substance use history frequently expressed a desire to limit interactions with her close knit “street family” because their frequent drinking and drug use threatened both the participant's sobriety and current housing placement.

“*It's particularly people that I hang with of my street family, the ones I've been hanging with and they do drink a lot… You know, I'm doing what I have to do. I'm staying away from, you know, people, places and things” (V-110, Chronic)*

Although this was something she felt like she needed to do, it was also difficult for her to distance herself from this part of her social network. During the study period she was often isolated in her room at the shelter. Her emotional state, as well as her health, fluctuated a lot during the study period, and often from one interview to another.

Finally, the contexts within which participants were living put them in social relationships that were often described as unhealthy and challenging. Staying in shelters or residential programs meant that a person's de facto social environment was comprised of other individuals they had not have chosen to be with. Many reported tense relationships with the peers in their shelter or transitional housing program. Concerns about violence, theft, and exposure to drug use frequently reported.

“*I come to this building… I wasn't planning on it being – I'm surrounded by addicts. I'm surrounded by drunks. I'm surrounded by everybody using, actively using stuff… that's why I'm trying to get out of there as fast as I can.” (V-102, Chronic)*

The desire to leave the shelter and move to a stable, individual housing unit, without substance use exposure, was frequently expressed by participants. Participants also reported feeling a lack of meaningful social attachment to many people in their lives, including social service workers who were supposed to help them. At the same time, some participants expressed concerns with feeling lonely and isolated when they are finally able to leave their transitional housing or treatment program. As one participant noted, “*I've seen some get up off the streets and get an apartment and die because of loneliness.” (V-315, At-Risk)* It was not uncommon for participants to express very different opinions about their readiness and confidence to move into more stable housing throughout their engagement in the study.

Finally, a few participants discussed their relationship with social and health professionals that provide some kind of support in their lives. Most felt that these relationships were relatively distal and inconsistent. The COVID-19 pandemic likely played a role in this, as restrictions on in-person meetings, requirements for social distancing, and precautions taken when someone was ill (or potentially exposed) disrupted preferred ways of connecting and communicating. Some engaged in services through telehealth, or virtual care. Although convenient, the structure of virtual appointments led many to feel like their providers did not know them or understand their circumstances. A few participants also talked about the stresses posed by reduced access to social and medical services during the pandemic, leading people to feel frustrated, trapped in unhealthy or unsafe living circumstances, and unable to get the kinds of support they needed.

### Implications for phase 3 pilot

#### Content

Findings from this formative phase suggest that social relationships are critical to ask about frequently through a mobile app. We observed that some relationships change relatively quickly, especially romantic partnerships and acquaintances within temporary housing programs. Social relationships are also not perceived to be consistently positive or negative but may fluctuate in relationship to a participants' personal goals, health status, and behaviors. Finally, variation in perceptions of one's social circumstances highlights the need to not make assumptions about size and composition, especially in relation to social support. Questions about preferences and satisfaction with social network are needed in order to understand patterns in communication.

#### Approach

Nearly all participants in both phases of the study emphasized the importance of meeting participants face to face when the study begins and spending time building rapport and trust with them. Their willingness to engage and provide honest answers hinges to a large extent on whether or not they can formulate a sense of trust in the researchers and believe in their motives. As one participant who took part in the Phase 1 ethnographic interviews explained,

“*Talking to you and meeting you, it makes it easier, now we actually had a face-to-face. So, now when I do talk to you [by phone], I know who I'm actually talking to, to give you the feedback that you need instead of some person just sitting on the computer.” (V-102, Chronic)*

Most participants placed a high value on in-person encounters. Although the goal of Phase 3 is to use smartphones for data collection, findings highlight the need to nest this data collection effort into a broader approach that includes taking time to get to know participants and their current circumstances before launching the smartphone data collection effort.

#### Design

With permission from participants, we will be leveraging smartphone capabilities to track patterns in communication with others through phone calls and texts. The app will be programmed to encrypt numbers so they are not traceable to individuals. As noted above, there will be a need to gather other information regarding preferences for size and composition of social networks and satisfaction with support provided to assist with interpretation of communication patterns.

### Income

The majority of participants reported little monthly income during the study period. About two-thirds of participants relied to some extent on military service benefits (e.g., physical or mental health disability stemming from time in service), with half relying solely on these benefits. A few relied on state disability benefits. One person held a full-time job at a VA hospital throughout the study period. Although he had a steady income, he depended on subsidized housing vouchers to make ends meet in a high-cost area. A few also talked about work they did in the informal economy, such as asking for spare change or selling alcohol, cigarettes, or drugs.

Most participants relied on public benefits for some or all of their monthly income. Over the course of a month, fluctuations in money contributed to feelings of stress, anxiety, and perceived loss of control over one's life. In particular, the beginning of a month meant that participants had money to pay for public transportation to leave the dense urban area they were living or to pay for a hotel room for a night or two to take a break from shelter life. As the month proceeded, money dwindled and participants had to rely more on public and social services to meet their basic needs. These fluctuations in income were common. We observed and participants reported that they felt more depressed, angry, and frustrated near the end of each month. In situations like this we observe that participants were less likely to be able to cope with unexpected events or flashpoints when they occurred, which could lead to unanticipated setbacks or transitions.

### Implications for phase 3 pilot

#### Content

In baseline interviews we will ask open-ended questions to gain an understanding of the amount and type of income participants typically rely on each month. We will ask about the extent to which they feel this income allows them to meet their needs. We will explore other resources or services that provide assistance. This baseline understanding will help us learn about fluctuations in income over the course of a month. A few times a week we will ask questions through the app about work (e.g., type) and perception of having enough income to meet one's needs. Asked repeatedly over the course of a study period, we will be able to explore how changes in income and perceptions of adequacy of income are related to mood, stress, depression, substance use, and other factors that can influence housing stability.

#### Approach

Given that there are a variety of income sources (e.g., benefits, formal wage labor, informal or illicit labor) that participants may rely on, it will be important for us to be inclusive in how we phrase our question(s). Concerns with confidentiality and privacy have prompted discussions about what is important to learn about income and work. We tentatively decided to ask about participants' engagement in work each week and whether or not the work was for: (a) money, (b) trade or barter, (c) without pay (e.g., volunteering), (d) did not work, and (e) prefer not to answer.

#### Design

With the smartphone-enabled data collection app we are able to program questions about income and work that might have daily and weekly fluctuations. For example, at the end of each night we are considering asking a single question about whether a participant worked that day. On a weekly basis, we can explore activities that might occur over a longer period of time, such as looking for work or participating in a work-training program. Total income and perceptions of adequacy of income to meet needs can be asked at random times throughout the study period and programmed to capture variation across a given month.

### Factors that may influence smartphone-enabled data collection

In addition to identifying key elements in the day-to-day lives of veterans experiencing homelessness, we sought to understand the feasibility of collecting research data *via* a smartphone app. Nine of the ten individuals who enrolled in the Phase 1 study and all nine individuals who participated in Phase 2 focus group discussions had their own smart phone. The veteran who did not have a phone upon enrollment in the study purchased phones somewhat regularly. However, he frequently damaged them when frustrated or mad. This veteran was provided two mobile flip phones by the research team to take part in the study. He damaged the first one shortly after our first interview and was provided a second one. After the second interview we lost communication with him.

Among those who had mobile phones, there was wide variation in how they were used in everyday life. On one end of the spectrum, a few participants indicated that they use the phone primarily to make phone calls and occasionally check email. These individuals tended to be older and more likely to report that they do not know how to use their phones to the full extent.

“*That there're no classes for the phone. That really ticks me off. How are you supposed to learn – like, I bought this phone, and it didn't even come with directions.” (V-104, Chronic)*

In addition to knowledge about how to use their phones, a few participants called our attention to the importance of language when introducing a study that involved smartphones and applications. For example, when asked about what kind of applications or “apps” he used on his phone, one participant indicated he did not know what an app was. Upon explanation, he told the interviewer that he had always referred to the app icons on his phone as “*little squares” (V-102, Chronic)*.

A few participants who described more limited uses of their phone noted physical challenges that impeded use. One person had limited dexterity with several fingers. Performing certain functions, like typing texts or emails, was reported to be difficult given the small screen and keyboard. Others had impaired vision that made it difficult to see small font and images.

On the other end of the spectrum, some participants consider their phone a lifeline in the world. They used it to stay in contact with people, including social and professional relations, for telemedicine appointments, geographic navigation, to identify services and resources, and for entertainment. For these individuals, their phones were critically important. “*Staying in communication depends on me having my phone, you know*?” *(V-314, At-Risk)*. One participant described the anxiety he felt when he temporarily lost his phone:

“*[At] the time I had my team or workers, my social worker, my therapist, my housing worker, my case workers, everybody in my phone was helping me to get where I'm at today and now I had to reestablish all my phone numbers on my finder and got my team back together. I was having an anxiety attack. Without my phone I was literally, I didn't realize how much I needed it.” (V-102, Chronic)*

The loss or theft of a phone, which was commonly reported, could mean the loss of important connections, setbacks in progress toward housing, and missed health or social service appointments. Loss of a phone is another type of flashpoint that can have ripple effects. In general, those who reported having their mobile phones lost or stolen in the past have been successful in obtaining new ones relatively quickly and tend to have access to support to get them back “up and running” with their digital connections. Some did rely on email or social media apps that they were able to access through a computer to stay in touch with others when they were without a phone.

The issue of trust emerged again in conversations about technology use and the potential of collecting data through a smartphone app. Many participants had smartphones that were free or subsidized through a governmental program (nicknamed “Obama phones” after the US President at the time of a policy to subsidize phone access). They had inexpensive phone plans. However, these lower cost options had drawbacks. Many reported getting a lot of spam texts, phone calls and emails. Repeated spam made it difficult to discern what incoming information was trustworthy.

“*Anybody who texts me, how do I actually know who's on the other end? How do I actually know the person is really sincere with what they're saying?...” (V-102, Chronic)*

These types of communications heightened concerns with surveillance and lack of privacy among some. For others, it meant they only responded to messages from recognizable numbers (i.e., in their contact list).

Finally, we explored perceptions of using additional features on a smartphone, such as global positioning systems (GPS) and phone logs to gain a better understanding of participants' mobility (i.e., how far people travel each day), activity space (i.e., types of places people go), and social contacts. Participants in the Phase 1 ethnographic interviews raised fewer concerns about GPS and phone log features. This may be due to our asking specific questions about the use of these features near the end of our data collection period, after we had repeated encounters with them. Focus group participants had more questions about why this information was needed, how much detail would be transmitted, and who would be seeing the data. Concerns were raised about the potential use of this information to track or monitor drug deals or other illicit activity. Of note, concern was anticipated primarily when someone was active in their drug use.

### Implications for phase 3 mobile app

#### Content

These findings did not have major implications for the content of our survey questions. However, we will incorporate a series of questions into our baseline interviews to learn about participants' comfort with and uses of their smartphone. This information will be useful to members of the research team to inform the type and extent of training that participants may need to participate in the app-based data collection.

#### Approach

Exploration of participants' use of and concerns about their smartphones over time highlighted a number of important considerations for how we approach enrollment in the Phase 3 pilot study. Clear communication about the purpose of the study and what participation entails will be important to enrolling people who are interested and willing to provided data through an app. While this is a required ethical practice, we think that this information needs to be shared before the formal informed consent process. For example, when introducing the purpose of the study at the initial recruitment phase, research members may need to use visual aids, such as showing example questions on a smartphone and pictures of activity spaces generated from GPS location data, so that potential participants have a good understanding of the study. If they remain interested, then a formal informed consent process can begin. Through this process we need to reiterate the purpose of the pilot study, how the data will be used, who will have access to their data, and how privacy and confidentiality will be maintained. Although this is standard ethical practice, the novel approach and time intensive nature of the study needs to be explained thoroughly prior to enrollment. This may also reduce the risk of recruiting people who are not a good fit for this phase of the study (i.e., are reluctant or opposed to sharing personal information through an app).

With the high level of theft and phone loss reported among participants, it is important to create ways for participants to stay engaged with the study. For example, we will have one research team member be the primary point of contact for the study. His contact information will be sent in email, text, and hard copy form. Participants will be asked to contact him with any questions or if they have lost their phone. We are also considering creating a way for people to respond to questions through other means, such as a tablet or link to a website. Finally, upon enrollment we will also ask participants to inform us of how to reach them if they have not responded to survey questions for a week. This will allow us to connect with them to learn about their situation and how best to get them re-engaged (if interested).

#### Design

Concerns with high volumes of spam communication, privacy and mistrust have highlighted the importance of branding the smartphone app and making it very clear when push notifications or requests to respond to daily or weekly questions are from the study. In a future focus group, we will provide several different logos and ask participants to identify which one is preferable. This may help participants decipher between a trusted communication and an unknown one.

Another design feature that was recommended by participants was the ability to make decisions about what information to share with the research team. For questions, they recommended an option to “refuse” or “decline” a response. For GPS data transmission, they recommended transmitting periodic requests to continue to sharing GPS location data or, if location data was turned off, to request turning it on again. We are considering a similar approach to requesting access to phone logs. This provides participants with the ability to know what personal data the smartphone app is accessing and allows choice over what and when to share.

“*… as long as you have that prompt where you can say yes and no [for sharing GPS data], I mean, everything is fine.”* (*V-316, At-Risk)*

Providing choices like this will allow the study team to learn more about the features of smartphone data collection that are acceptable to participants. If enough participants provide consent to use these other types of data, we will have the opportunity to determine their value in understanding dynamic factors that contribute to housing instability and homelessness.

## Discussion

Ethnographic research guided by a user-centered design framework provided our research team with a depth of information to inform the content, approach, and design of our planned smartphone-enabled pilot study with veterans experiencing and at risk of homelessness. Frequent data collection using questionnaires distributed *via* a smartphone app at regular intervals may provide information traditional survey research have been unable to do—identify the sequence of events and experiences that precede and follow the transition from one housing status to another. Such an effort may facilitate early identification of potential problems, offering service providers a chance to prevent or intervene quickly enough to mitigate them. The planned pilot study will explore the feasibility and acceptability of using smartphone apps to identify or detect near real-time events, activities, moods, and triggers that presage negative outcomes such as housing instability, loss, or serious health events. To our knowledge, this will be among the first test of active and passive smartphone-enabled data collection applied to the study of homelessness among veterans.

While our sample of veterans experiencing homelessness was relatively small, we were able to gather extensive, longitudinal data about their daily patterns. We collected up to 30 days of data across ten participants, providing a depth of information about fluctuations in their daily lives. The participants represented a range of housing situations, including transitional housing, residential treatment programs, shelters, and one individual was street homeless. More than half of participants had a major transition in housing during the 4–6 week study period. This finding lends support for our initial study design which aims to develop and test a smartphone app over a limited period of time for “proof of concept.” Further, the ethnographic data provided insights into the types of transitions that might be made, catalysts for these transitions, and potential fluctuations we might detect based on season and time of month.

Similar to other studies conducted with individuals experiencing homelessness (36, 38, 59), we found near ubiquitous ownership and use of smartphones among participants in our sample. The one individual who did not have a phone at the time of enrollment in the study, reported recent and frequent smartphone ownership (reportedly buying 40 phones a year, all of which were lost or damaged). Although phone theft or damage was frequently reported, the majority of participants secured new phones quickly. For many, it was their “lifeline” to family, friends, healthcare providers, and social services. Many used their phones to access needed medical and social services, navigating from one location to another, and for entertainment (e.g., games, movies). Technological competency did vary however, with older veterans less likely to report using a broad range of functions on their smartphones and more likely to report challenges with use (e.g., dexterity to type, impaired vision to read small fonts).

A key lesson learned about our *approach* to implementing the Phase 3 pilot study is the need account for a range of technology user expertise by tailoring an initial training on how to answer questions through the smartphone app. Attention to the wide variation in digital literacy will be important for consistent participation (38, 60–62). We are considering incorporating a series of digital literacy screening questions when introducing the study so that the research team is able to tailor training on how the smartphone app functions and how to provide responses to different types of questions. We expect that some individuals will be familiar with how apps work and quickly know how to use the app. Others may need an overview of how apps work, including where and how information is stored, how to adjust settings to increase ease of use (e.g., increase font size), and how to answer each type of question. In addition, we will provide a single point of contact on the study team who is available to answer questions and provide technical support. Investment in this type of tailored training and on-going support may help reduce frustration with technology. Individualized training can also contribute to building trust and rapport with participants so they feel comfortable reach out for assistance. Notably, the response to our questions about trust varied between the Phase 1 participants who got to know their assigned researcher, while the Phase 2 focus group participants were more circumspect.

With respect to *design* of the app, findings highlighted the need to offer choices that allow participants to participate. For example, we can design options for font size and help participants select one that is best suited for them. We are also considering offering “voice to text” options for open-ended questions to accommodate participants with limited dexterity or comfort typing on a smartphone keyboard. Designing the app to include choices to opt in or out of data requested was highly recommended among participants. Issues related to privacy and trust were of concern, particularly with respect to questions about sensitive issues (e.g., drug use) and passive data sources, such as GPS location data and call logs. Providing participants with an option to turn off or refuse to transmit information may improve willingness to participate in the novel research study. In addition, we are developing robust procedures to protect privacy and data security of any data submitted or tracked. Our *approach* to explaining these safeguards and providing adequate training on how to opt in or out of a data request will be critical.

The dynamic fluctuations in the lives of participants over the course of the Phase 1 study highlighted the potential value of using a smartphone app to collect information about the day-to-day experiences that influence transitions in housing and health. However, building a digital health tool offers very little if people do not use it or the quality of response data is poor. For people experiencing homelessness, lack of trust in people generally, and certain entities in particular (e.g., “government”), can be a major barrier to engagement in many services (9). Trust was found to be important to decisions about participating in a study like ours that aims to collect personal information *via* a smartphone app. Attention must be paid to how creators of digital health tools and/or those who want to implement them can build a sense of trust with a potential tool user. In our study, our team discussed the need to meet participants in person to introduce the study and to be prepared to offer one or more introductions and enrollment sessions. Clearly communicating the purpose of the study and how we are safeguarding the information they share through the app will be critical to participation. Participants will need to have a clear understanding of the purpose of study and the rationale for different types of questions we might ask (i.e., “We ask about substance use because it may mean there will soon be changes in social relationships and housing”). Importantly, they will need options for what to share and when to share data.

Use of a smartphone app for data collection has some limitations, especially in regard to the number of questions that can reasonably and feasibly be asked at a given time. As with any questionnaire, this means that it is important to understand what is relevant and meaningful to ask about (i.e., *content*) and the frequency with which to ask. Our ethnographic data provided a number of examples of how transitions in housing are rarely linked to a single event. For many, conflicts in relationships, unmanaged health conditions, and repeated challenges securing documents or completing paperwork for housing subsidies were often catalysts for changes in mood, which sometimes led to substance use as a coping mechanism. Some temporary housing environments were also stressful and unpredictable, with high levels of substance use and violence reported. For some, the options were to isolate as best they could, or join others to get by; these options had pros and cons for different participants which would be important to discern. Frequent data collection allowed us to see changes in mood and health over the course of a month as a person's fixed income dwindled. Options to take a break from a shelter by staying in a hotel or to exercise choice over their meals were greatest at the beginning of the month when many received benefit payments. As income declined over the month, stress, anxiety, and depression seemed to increase. These changes in mood affected participants' response to key events, such as an altercation in a personal relationship or bureaucratic barrier. Sometimes this influenced decisions to use drugs and alcohol, which also had variable impacts on participants' housing, health, and general sense of well-being.

While the ethnographic methods employed during this formative phase of our pilot study offered rich and nuanced insights to guide the content and design of the smartphone data collection app, there are a number of limitations worth noting. First, there are limitations related to the study sample. Given the intensity of data collection (volume and period of time) and need for rapid analysis to inform subsequent phases, formative data could only feasibly be collected from a small sample of Veterans experiencing homelessness. The research team understands the heterogeneity of the population of veterans who are homeless or at risk of homelessness. However, we had to make decisions about what qualities or perspectives would be important to understand for the pilot study, which is a “proof of concept” that near-real time data can be collected from people experiencing homelessness through a smartphone app. Drawing on our prior research with veterans with unstable housing and conversations with our veteran research consultants, we opted to prioritize sampling based on variation in homeless experiences; our goal was to recruit Veterans whose housing situations ranged from at risk to long-term and chronically homeless. We anticipated that these different circumstances may influence access to and use of smartphones. Although we did not specifically recruit for other types of diverse experiences, our sample reflected some, but not a full range of diversity among this population in the metro area, including 40% who identified as Black and 10% as female. We also had a range of age groups, although the majority were over the age of 50 years. To our knowledge, our sample does not include veterans who identify with racial/ethnic groups other than White/Caucasian or Black/African American or as gay, lesbian, bisexual, transgender, or non-binary. The sample also does not account for variation in experiences with homelessness based on geographic location. It is likely that transitions in housing and decisions about housing more generally are affected by cost of living and seasonal variations in weather. The metro area is both expensive and seasonally cold for approximately half of the year, both of which influenced housing decisions in our sample. Diversity with respect to race, ethnicity, gender, age, and geographic location are important to understand before we scale up a smartphone-enabled study approach, particularly as they influence our thinking about what is important to ask about (i.e., content of our questions) and how best to ask (i.e., approach).

A second limitation related to our sample is with the narrower diversity of focus group participants in Phase 2. Our intention was to leverage our success with recruiting veterans from one shelter (NECHV), which housed veterans with recent and chronic homeless experiences, to provide rapid feedback on specific questions related to approach and design, such as how to explain our interest in GPS location data and call logs. The sample did not include one of our three priority groups, veterans at risk for homelessness. This was a difficult group to identify and recruit for Phase 1 interviews. This perspective is missing from the initial focus groups. As we move into the usability testing phase, greater attention to including representatives from all three groups will be important.

Finally, we offer our perspectives on a challenge as researchers gathering and analyzing data from individuals who have a broad range of experiences and opinions related to using smartphones to collect data in near real time (63). This challenge is what Oliver et al. (63) refer to as “the dark side” of co-design work. As with any user-centered design approach, our team must decide how to prioritize the range of events, experiences, physical and emotional states that participants shared as being important in their everyday lives. We must also manage divergent opinions about approach and design features. There is no playbook for how to approach this variation. Our qualitative team has spent hours reviewing notes and transcripts, identifying salient and unique themes. We have shared these insights with the broader team, some of whom are focused on survey development and others on app design. Decisions about what questions to ask, design features, and approach are necessarily iterative, tacking back and forth between figuring out what content is important, what is reasonable and feasible to ask in an app format, and what options are needed to increase acceptability. The work requires a multi-disciplinary team to move a project like this from concept to product.

## Conclusion

Our findings highlight the value of bringing together ethnographic methods and user-centered design frameworks to develop digital health tools. Smartphones offer a variety of benefits for people experiencing homelessness, including connecting them to people and services (35, 64). The ubiquity of smartphones in people experiencing homelessness potentially present novel methodological options for research. This formative study is part of a larger research agenda to understand the extent to which smartphones can also be used to facilitate a variety of different types of data collection from people experiencing homelessness. Collecting data *via* smartphones may allow us to gather almost real time data, which may support interventions to intervene in a timely manner if we can identify key indicators that lead to a housing transition. If we are able to demonstrate the feasibility of smartphone-enabled data collection, additional formative research with an expanded diversity of veterans experiencing homelessness is needed. This includes veterans who are vulnerable to homelessness or may have different experiences related to homelessness because of their sexual and gender identity, race and ethnicity, and geographic location. This is a critical step to take before launching a large-scale data collection effort using smartphone applications. Similarly, engaging a highly diverse sample of veterans in usability testing, which is our next phase of work, will be important to creating a relevant, user-centered digital data collection tool.

## Data availability statement

The datasets presented in this article are not readily available because this will need to be approved by the VA IRB, requiring a modification. Requests to access the datasets should be directed to DM: Keith.Mcinnes@va.gov.

## Ethics statement

The studies involving human participants were reviewed and approved by VA Bedford Healthcare System. The patients/participants provided their written informed consent to participate in this study.

## Author contributions

All authors listed have made a substantial, direct, and intellectual contribution to the work and approved it for publication.

## Funding

This research was funded by VA HSRD, Project # IIR-18-244.

## Conflict of interest

The authors declare that the research was conducted in the absence of any commercial or financial relationships that could be construed as a potential conflict of interest.

## Publisher's note

All claims expressed in this article are solely those of the authors and do not necessarily represent those of their affiliated organizations, or those of the publisher, the editors and the reviewers. Any product that may be evaluated in this article, or claim that may be made by its manufacturer, is not guaranteed or endorsed by the publisher.

## Author disclaimer

The views expressed in this article are those of the authors and do not necessarily reflect the position or policy of the Department of Veterans Affairs or the United States Government.
