# Supplementary material for: Longitudinal high-frequency ethnographic interviewing to simulate and prepare for intensive smartphone data collection among veterans with homeless experience
Source: Front Digit Health. 2022 Aug 12;4:897288. doi: 10.3389/fdgth.2022.897288 (PMC9411857; doi:10.3389/fdgth.2022.897288)
Supplement: Supplementary file 1 [file Data_Sheet_1.PDF]

Appendix 1: Interview Guides

**Phase 1 Interview Schedule**	
**Week 1**	Baseline
	Repeated Qualitative Inquiry- *Short*
	Repeated Qualitative Inquiry- *Short*
	Repeated Qualitative Inquiry- *Long*
**Week 2**	Repeated Qualitative Inquiry- *Short*
	Repeated Qualitative Inquiry- *Short*
	Repeated Qualitative Inquiry- *Long*
**Week 3**	Repeated Qualitative Inquiry- *Short*
	Repeated Qualitative Inquiry- *Short*
	Repeated Qualitative Inquiry- *Long*
**Week 4**	Repeated Qualitative Inquiry- *Short*
	Repeated Qualitative Inquiry- *Short*
	Final Interview
**Phase 2**	Focus Groups

INTERVIEW 1 – <u>Week 1 – at beginning of week 1 – Baseline</u>

TYPICAL DAY
1. What does a typical day look like for you?
2. What source(s) of income do you have?
3. Do you feel that your income allows you to meet your basic needs?
4. In what ways has COVID affected your routines and where you live? What it is like to live there?

HOUSING HISTORY
1. Where you are living now and work backwards from there, month by month.
2. What has your housing situation over the last 10 years?
3. Please think about your early adulthood (roughly ages 18-30). How would you describe your housing situation during these years?
4. How would you describe your housing situation while growing up?
5. What other things do you think are important for me to know or understand about your experience with housing?

SOCIAL RELATIONSHIPS
1. Who are the people in your life who support for you right now? How long they have been in their lives?

Person 1_____________________________ Person 5_____________________________
Person 2_____________________________ Person 6_____________________________
Person 3_____________________________ Person 7_____________________________
Person 4_____________________________ Person 8______________________________

2. To what extent do you think you have the kind of support from others that you want right now?
3. What do you think influences the social and emotional support/companionship you have right now?

USE OF TECHNOLOGY

<u>Mobile Phones</u>

A mobile phone is any phone that you can take around with you. It doesn't necessarily need to do fancy things like connect to the internet.

1. To get us started, do you currently own a mobile phone or have access to one?

☐ Yes, I own one

☐ Yes, I have access to one when I need it (but not my own)

☐ No → Have you ever had your own mobile phone or access to one? Why don't you have one now?

IF NO TO ALL, then SKIP to question 22.

IF YES → Is the phone you have access to a smartphone (i.e., iPhone, Android, Blackberry, Windows Phone)?

☐ Yes

☐ No

☐ Not sure / don't know

2. How many different mobile phones have you had over the last 6 months? _______
3. How easy or hard is it for you to pay for the service for your mobile phone?
4. How often do you share your mobile phone with another person?
5. How often do you borrow a mobile phone from someone else?
6. How often would you say you use your phone (or someone else's phone) on a typical day? (# of times/# of minutes/hours/day) _____________
7. What types of things do you mainly use your phone for?
8. Could you tell me about your use of texting?
9. How much do you depend on your phone to do things that you want and need to do every day?
10. Do you have concerns about sending or receiving information through your phone?
11. How would you describe your ability to use your mobile phone for typical day-to-day purposes?

☐ I experience little to no challenges in completing any tasks on my phone

☐ I experience some challenges in using my phone, but I am still able to do what I need to do

☐ I experience significant challenges in using my phone that impact my ability to do what I need to do

☐ The trouble I experience in using my phone keeps me from using it regularly

☐ I feel a different way (please describe)

<u>Internet</u>

Now I have questions about using the internet more generally.

12. About how often do you use the internet?

☐ Several times a day

☐ About once a day

☐ 3-5 days a week

☐ 1-2 days a week

☐ Every few weeks

☐ Less often

☐ Never

☐ Don't know

13. Do you mostly access the internet using:

☐ A mobile phone

- ☐ A computer (could be a desktop, laptop, or tablet computer)
- ☐ Both equally/depends

14. What else do you think is important for us to know or understand about how you use technology, like mobile phones and the internet?

INTERVIEW – <u>Weeks 1-4 – beginning and middle of each week – Repeated Qualitative Inquiry - Short</u>

(5-10-minute check-ins between the weekly longer interviews)
I just want to check in and see how things have been going for you the past couple days. This should just take about 10 minutes.

INITIAL CHECK-IN

1. How are you doing? Has anything new happened that since we last spoke that you'd like to share?
2. How are you feeling today?
    a. How are you feeling physically? [On a scale from 1 to 5, with 1 being the worst you have felt and 5 being the best, how are you feeling physically?]
    b. How are you feeling emotionally? [On a scale from 1 to 5, with 1 being the worst you have felt and 5 being the best, how are you feeling physically?]

HOUSING / MOVEMENT / ACTIVITY SPACE

1. How did you spend your day yesterday? Beginning with the time that you first woke up, what did you do? [Listen for places that she/he went to and probe for information about why they went to place X, what they did there, who they were with)
2. How was your night last night? Where did you sleep?
    a. [If same place:] How was that for you?
    b. [If different from where they were the last time you spoke with them ask:] How that was? And what led to you staying there rather than the place you were staying a couple days ago? How long do you think you will stay there?

SERVICE USE

1. Since we last talked, have you received any health-related services like a clinic visit for physical health or mental health, a hospital stay, emergency room visit, inpatient or residential detox program, or outpatient substance use treatment (including methadone)?
2. And what about other services such as legal services, job training or placement, AA/NA, foodbank, etc. Have you taken part in any services like these over the last few days?
3. Is there anything else that is important to know or that you would like to share about how things are going for you?

Thank you so much for talking today. I'll be talking to you again __________ (Date/Time, if known).
I look forward to it.

INTERVIEW – <u>Week 1 – end of week 1 – Repeated Qualitative Inquiry - Long</u>
Focus on Health

CHECK-IN QUESTION [interviewer: bring previous interview synthesis to interview for reference]
1. We talked [a couple / several] days ago. Before talking about your health, I just want to check in and see how things have been going for you this past week. How are you?
- What have been some of the highlights or good things that have happened this past week?
- What has been challenging?
- How have you been feeling? (e.g., happy, anxious, depressed, pain etc.)?

2. Where are all the places you have stayed/slept over the last week?
- How was this for you?
- How did you come to stay at [location currently staying]?
- How long do you think you will be at [location currently staying]? What makes you think it will be [length of time]?
- What prompted you to leave [place they were staying]?
- What do you see as the important events that led you to coming to stay at [location where currently staying]?
- What are the good things about living at [location currently staying]?
- What are the hard or challenging things?

3. What <u>services</u> have you used over the last few days? (e.g., social or medical services – including ER; mental health or substance use services; employment or training services)
4. How has COVID impacted where you lived, where you went, or what services you used over the last week?
5. Have you had any challenges with your mobile phone (e.g., keeping it charged, keeping service plan and/or minutes, lost or stolen, used by others, etc.)? If so, what challenges have you had? How have these challenges impacted your ability to get things done this week and/or to communicate with others when needed?

HEALTH
1. How would you describe your health right now?
Now I'd like to ask you about your physical health.

1. What kinds of physical health conditions are you living with?
- How long have you been living with [each condition]?
- Where are you getting healthcare to address these physical conditions? What do you think of the healthcare you are getting?
- How are you managing these physical health conditions right now?
- [*If more than one*] Which one is the most difficult to manage? Why?
- How do these physical health conditions impact your day-to-day life?
- More specifically, how do you think these physical health conditions have impacted your housing over the last few months? Please explain.

2. Now I'd like to talk about your mental health. By mental health I mean how you are feeling emotionally and spiritually. How would you describe your mental health right now? (or how you are feeling emotionally and spiritually?)
- What kinds of mental health conditions are you living with?

- Where are you getting mental health care right now? What do you think of the mental health care you are getting?
- How are you managing your mental health conditions right now?
- [If more than one] Which one is most difficult to manage? Why?
- How does your mental health impact your day-to-day life right now?
- How do you think your mental health has impacted your housing over the last few months? Please explain.

3. Now I'd like to talk about use of substances, such as drugs, alcohol, and over the counter and prescription medications used in ways not described on their packaging or as prescribed by a doctor. How would you describe your use of substances right now?
    - Have you ever felt like, or been told, that you have a problem with drugs or alcohol? If yes, which ones? If no, skip to question about current substance use
        - Which substances are or have been most concerning for you? How long have they been a concern for you (and others in your life)?
        - What kind of substance use treatment or support are you engaged in right now, if any? What do you think of this treatment/support?
    - What substances (drugs and/or alcohol) are you currently using, if any?
    - How does your substance use impact your day-to-day life right now? What are the things that make it hard to manage your substance use? What helps you manage your substance use?
    - How do you think your substance use has impacted your housing over the last few months?
4. What medications are you taking on a regular basis (e.g., daily or weekly), (if any)? How easy or hard is it to take them the way the doctor prescribed them?
5. What else is important for us to understand about your health, particularly as it relates to your housing?
6. Thinking of the health information you have shared with me today, would you share this kind of information with researchers if they collected it through your mobile phone, like by text messaging, or survey questions that you answer on your phone? Why or why not?

INTERVIEW – <u>Week 2 – at the end of week 2 – Repeated Qualitative Inquiry - Long</u>
Focus on Service Utilization

CHECK-IN QUESTION [interviewer: bring previous interview synthesis to interview for reference]
1. We talked [a couple / several] days ago. Before talking about services, I just want to check in and see how things have been going for you since we last talked. How are you doing?
  - What have been some of the highlights, or good things that have happened since we last spoke?
  - What has been challenging for you?
  - How have you been feeling? (e.g., happy, anxious, depressed, pain etc.)? Probe for why.

2. Where are all the places you have stayed/slept over the last couple of days?
  - How was this for you?
  - How did you come to stay at [location currently staying]?
  - How long do you think you will be at [location currently staying]? What makes you think it will be [length of time they said]?
  - What prompted you to leave [place they were staying]?
  - What do you see as the important events that led you to coming to stay at [location where currently staying]?
  - What are the good things about living at [location currently staying]?
  - What are the hard or challenging things?

7. What <u>services</u> have you used over the last few days? (e.g., social or medical services – including ER; mental health or substance use services; employment or training services)
8. How has COVID impacted where you lived, where you went, or what services you used over the last week?
9. Have you had any challenges with your mobile phone (e.g., keeping it charged, keeping service plan and/or minutes, lost or stolen, used by others, etc.)? If so, what challenges have you had? How have these challenges impacted your ability to get things done this week and/or to communicate with others when needed?

SERVICES TO HELP MEET NEEDS
1. Next I'd like to ask a few questions about the kinds of services you are taking part in right now. What kinds of services are you currently using or engaged in right now? *Write down each one mentioned.*
  - What does [type] service provide?
  - How long have you been taking part in [type] service?
  - Where is [type] service located?
  - How often do you get services from this agency?
  - How do you usually get there?
  - How adequate do you think [type] service is in helping you get what you want/need? What are the good things about [type] service? What is challenging? What could be improved?

2. What kinds of services are you looking for or wish you had access to but do not have right now? *Write down each one mentioned.*
  - How do you think this/these services would be helpful for you?
  - How do you think they would impact your housing, if at all?
  - What problems or issues have you had in getting or using these services? What would be helpful in connecting you with these services (if appropriate/available)?

3. What else is important for us to understand about the services that are currently available to you or that you would like to access?

4. Thinking of the information about services you have shared with me today, would you share this kind of information with researchers if they collected it through your mobile phone, like by text messaging, or survey questions that you answer on your phone? Why or why not?

INTERVIEW – Week 3 – at the end of week 3 - Repeated Qualitative Inquiry - Long
Focus on Technology Use

Do you have any questions before we begin?
CHECK-IN QUESTION [interviewer: bring previous interview synthesis to interview for reference]
1. We talked [a couple / several] days ago. Before talking about technology use, I just want to check in and see how things have been going for you over the last week. How are you doing?
- What have been some of the highlights, or good things that have happened since we last spoke?
- What has been challenging for you?
- How have you been feeling? (e.g., happy, anxious, depressed, pain etc.)? Probe for why.

2. Where are all the places you have stayed/slept over the last couple of days?
- How was this for you?
- How did you come to stay at [location currently staying]?
- How long do you think you will be at [location currently staying]? What makes you think it will be [length of time they said]?
- What prompted you to leave [place they were staying]?
- What do you see as the important events that led you to coming to stay at [location where currently staying]?
- What are the good things about living at [location currently staying]?
- What are the hard or challenging things?

3. What services have you used over the last few days? (e.g., social or medical services – including ER; mental health or substance use services; employment or training services)
4. How has COVID impacted where you lived, where you went, or what services you used over the last week?
5. Have you had any challenges with your mobile phone (e.g., keeping it charged, keeping service plan and/or minutes, lost or stolen, used by others, etc.)? If so, what challenges have you had? How have these challenges impacted your ability to get things done this week and/or to communicate with others when needed?

TECHNOLOGY
1. In our first interview, you mentioned that you do/do not have your own mobile phone. Has this changed at all since we talked?
2. Have you had to replace your phone or get a new one? If so, why?
3. What are the kinds of things you generally use your phone for? What have you used it for in the last week? Probe for the following:
- Communicate with others? Who?

- Find resources? What kind?
- Navigation? Where to?
- Check email?
- Calendar/remember appointments? Which ones?
- Contacts?
- Apps that you use? (for fun, fitness, etc.)

4. How often do you use your phone during the day? (As an example, ask for how much they used it yesterday)

- About how often do you use your phone to send and/or receive text messages specifically?
- How easy or hard is it for you to send text messages? How easy or hard is it for you to receive text messages?

5. Where do you usually keep your phone?
6. What challenges do you face with your phone, if any? (e.g., keeping it charged, privacy, etc.)
7. In the last week, what other kinds of technology have you used that has been helpful for you?
8. Thinking of the information about your use of mobile phones that you have shared with me today, would you share this kind of information with researchers if they collected it through your mobile phone, like by text messaging, or survey questions that you answer on your phone? Why or why not?

INTERVIEW – **<u>Week 4 – at the end of week 4 – Final Interview</u>**

CHECK-IN QUESTIONS

We talked [a couple / several] days ago. Before I ask you the main questions, I just want to check in and see how things have been going for you these past few days. How are you doing?

- What have been some of the highlights, or good things that have happened since we last spoke?
- What has been challenging for you?
- How have you been feeling? (e.g., happy, anxious, depressed, pain etc.)? Probe for why.

1. Where are all the places you have stayed/slept over the last couple of days?
2. What <u>services</u> have you used over the last few days? (e.g., social services; medical services (including ER); mental health or substance use services; employment or training services)
3. How has COVID impacted where you lived, where you went, or what services you used over the last week?
4. Have you had any challenges with your mobile phone (e.g., keeping it charged, keeping service plan and/or minutes, lost or stolen, used by others, etc.)? If so, what challenges have you had? How have these challenges impacted your ability to get things done this week? How have they impacted your ability to communicate with others when needed?

FACTORS INFLUENCING TRANSITIONS IN HOUSING

1. Over the last few weeks, you have shared with me a broad range of life experiences, from early childhood through to the present. During our conversation, you have shared a lot about things that have had a big influence on your life, and particularly your housing. Some of the of the things I have picked up during our conversations is that [_________, ___________, and __________] have played a role in your housing situation over time. What do you think about my assessment?
    a. What else do you think has influenced transitions in housing throughout your life? [This could be from being housed to losing housing, or from not having housing to getting new housing.]
    b. Which of these things do you think are most important for your transitions into or out of housing?
    c. Interviewer: Select 3-4 influences noted above and ask the following question: You know yourself better than anyone else. When you think about how [____________________] has influenced your housing? What are the warning signs or triggers that indicate to you that you may be heading towards a change in housing? (What would be the signs or signals that you might be about to lose or change your housing?)
    d. What else have we not talked about that you think is important for us to know about your transitions in housing?

PERCEPTIONS OF STUDY

In this last set of questions, we are interested in hearing more about what it has been like for you to be a part of this project.

1. What has it been like for you to check in with someone like me (a researcher) on a regular basis this past month?
    a. What have been the good aspects of being involved in a study like this? What has been challenging?
    b. Is there anything you can imagine that would have made it easier for you and me to stay in regular contact?

c. Is there any other information we could have provided you at the start of the study that would have been helpful for you to know about the study? (This will be helpful for when we talk to other veterans who start this study in the coming weeks)

SHARING SURVEY INFORMATION WITH MOBILE PHONES

Next year we will create a mobile phone app—which is like a program that runs on your phone-- that will allow us to have veterans in our study answer questions on their phone instead of having in-person meetings or phone calls. We're thinking this would sometimes be a question or two done through text, and other times a longer survey with check boxes, drop down menus, or typed answers.

You have generously shared so much about yourself with me [and my colleagues].

2. What do you think it would be like to be asked to share this same kind of personal information with researchers through your mobile phone with an app or survey (without the live conversations with people)?
3. What concerns might you have if the mobile phone survey questions asked about things such as:
   a. Your mood, or your emotional state?
   b. Your feelings about how safe/stable your housing is?
   c. Your use of drugs or alcohol? (If you used, what kind/type, how often, etc.)
   d. Your use of services such as for healthcare or for housing?
   e. Your personal relationships?
4. Are there any of these things I just mentioned (mood, your housing stability, drugs/alcohol use, and use of services, etc.) that you would definitely <u>not</u> want to share via mobile phone survey, with a research team like ours?
5. Anything else you think we should/shouldn't ask?
6. What are some of the things you would recommend to people like us to help make Veterans feel comfortable sharing potentially personal information like this through their mobile phones?

FINAL WRAP UP QUESTIONS

1. Thinking back on your most recent Housing Transition can you remind me what caused that transition or why you made that move? What went well, and was difficult about it?
   a. Probes:
   b. How could it have been better?
   c. Services?
   d. People who could have helped?
   e. Concrete things? Moving help? Financial support for utilities or other expense?
2. What other recommendations or suggestions would you offer to us as we continue developing our study and learning about factors influencing housing among Veterans?
3. Given everything we've talked about these last few weeks - what else should I know? Or is there anything else you've thought out during our conversations that you think are important for me to know?

FOCUS GROUPS – **Phase 2**

1) GENERAL RESPONSE TO CELLPHONE STUDY. What are your general thoughts about taking part in a research study that asks you to complete short surveys on your phone every day or every other day?

- How would you want to be reminded about the surveys?
  - Probe: How does "The VA wants to hear from you" sound to you?
- How important would it be for you to be able to tell our surveys from other spam/scamming? Do you have trouble with spam/scamming often? What do you do to figure out which things are legitimate?

2) GPS SPECIFIC QUESTIONS. Another type of information we would like to collect is GPS/location information from your phone. We would use this information to better understand where people are, how they move around during the day and evening, and what services they are using.

- What do you already know about GPS / location services on your phone?
- What do you think it would be like to share this kind of location information with a research team? What kind of concerns would you have, if any?
  - i) Probe: Are you ok with sharing your location knowing we aren't interested in your personal location?
- We are planning to have an on-off button on the phones for GPS data – what do you think of giving people in our study the ability to turn those things on and off?
- We would also like to send people short surveys or information based on their location in the moment, such as if GPS showed you were at or near a VA medical center, sending you questions after you left asking what your experience was like. What do you think about this idea?

4) POTENTIAL FOR CALL/TEXT LOGS. We are interested in how much people communicate by text message and by phone call. What are your thoughts about researchers like us collecting information on phone and text message history—just the phone numbers, not the information in the phone call or the information in the text message? That information can often show things like whether a person is feeling social or not, and how many social connections they have.

a) Would you be OK sharing this information with researchers? What protections/assurances would you need to share these data?

b) We are planning to have an on-off button on the phones for this call data – what do you think of giving people in our study the ability to turn those things on and off?

c) Similar to the GPS data, we would also like to send people short surveys or information based on their calls or texts they make or receive, such as if you communicated with a VA provider you might get a short survey asking what your experience was like. What do you think about this idea?

5) SENSTIVE TOPICS. We would also like to ask about topics that are potentially sensitive, such as drug or alcohol use. Please feel free to answer these questions if they apply to you.

i) How comfortable would you feel providing information about drug or alcohol use in the past, such as the prior day or week (if it applies to you)?

ii) How different might you feel if you were asked questions about your drug or alcohol use at a time when you were actively using vs. a time when you were not using? [Explore]

iii) What would be your concerns about answering questions honestly about drug or alcohol use?

iv) What would your recommendations be for asking about drug and alcohol use in a way that helps people feel comfortable sharing this information?

When a Veteran enrolls in our study, we are hoping to provide them with a card (like a debit card or credit card) that we can add money to each week based on how many surveys the Veteran completes by cell phone or computer. So, for example, every time they complete a survey, money is added to their card. What do you think about this?

What kind of incentives for participation would be of interest to you?

- ☐ Gift cards after each short survey
- ☐ Payments automatically added to a study debit card after each short survey
- ☐ Free phone from the study
- ☐ Cell phone bill paid for certain term (e.g. 6 months).
- ☐ Other?
